# Meta-Analysis of Vitamin D Receptor Gene Polymorphisms in Childhood Asthma

**DOI:** 10.3389/fped.2022.843691

**Published:** 2022-04-01

**Authors:** Yong Zhou, Sheng Li

**Affiliations:** Department of Pediatrics, Yancheng Maternal and Child Health Care Hospital, Yancheng, China

**Keywords:** vitamin D receptor, polymorphisms, childhood asthma, systematic review, susceptibility

## Abstract

We conducted the systematic review to investigate the potential relationship between the vitamin polymorphisms of D receptor (VDR) gene and childhood asthma. Relevant studies researching on VDR polymorphisms and asthma susceptibility were searched throughout Embase, PubMed, China Science and technology journal database (CQVIP), etc. till 12 April, 2021. We calculated the pooled odds ratios (OR) and its 95% confidence interval (CI) using RevMan 5.3 software and Stata 11.0. FokI (rs2228570) could significantly affect childhood asthma risk across co dominant model (Ff vs. FF: OR (95%CI) = 0.82 (0.65, 1.02), *P* = 0.071) and dominant model (ff+Ff vs. FF: OR (95%CI) = 0.77 (0.63, 0.95), *P* = 0.016), especially among Caucasians in additive model (f vs. F: OR (95%CI) = 0.63 (0.43, 0.92), *P* = 0.015) and dominant model (ff+Ff vs. FF: OR (95%CI) = 0.67 (0.51, 0.88), *P* = 0.004). TaqI (rs731236) was significantly related with childhood asthma in additive model (t vs. T: OR (95%CI) = 0.45 (0.23, 0.89), *P* = 0.022), co dominant model (Tt vs. TT: OR (95%CI) = 0.36 (0.17, 0.77), *P* = 0.009), and dominant model (tt+Tt vs. TT: OR (95%CI) = 0.36 (0.15, 0.87), *P* = 0.024) among Asian, as well as population-based subgroup in co dominant model (Tt vs. TT: OR (95%CI) = 0.53 (0.31, 0.94), *P* = 0.029). However, no evidence supported the role of ApaI (rs7975232) and BsmI (rs1544410) polymorphisms in childhood asthma. FokI and TaqI polymorphisms were found to be related with the susceptibility of childhood asthma. However, it seems that ApaI and BsmI polymorphisms are not related with childhood asthma susceptibility.

## Introduction

Asthma is recognized as a chronic heterogeneous respiratory disease, which has characterized by airway inflammation and hyper-responsiveness, and the disease affects more than 300 million people worldwide, especially among children (1). The incidence, morbidity, and mortality related with asthma was influenced by several potential risk factors such as environmental factors (2), infancy microbial, biome influences, and genetic background, including vitamin D receptor (VDR) gene (3).

Vitamin D has been shown to have potent immunomodulatory properties, and Vitamin D correlated with the regulation of adaptive and innate immune function through VDR (4). Recently, increasing evidence researched on the effect of vitamin D in asthma and demonstrated that the severity of symptoms was related with vitamin D deficiency (5, 6). Among VDR polymorphisms, four SNPs, including BsmI (rs1544410), ApaI (rs7975232), FokI (rs2228570), and TaqI (rs731236) have been widely researched (7), but the relationship remains inconsistent. For example, the meta-analysis by Makoui et al. showed a statistical significant association between asthma risk and TaqI SNP (7). However, the systematically review by Zhen et al. showed no association between TaqI SNP and asthma risk (8). Additionally, thereafter, some new studies have been published (9–13).

Thus, it is necessary to update the report based on the previous results of researches to further explore the potential role of VDR genes polymorphism in childhood asthma susceptibility. Then, we designed the meta-analysis and explored this relationship in different races and source of controls. Finally, our data demonstrated that FokI and TaqI polymorphisms might be associated with childhood asthma susceptibility. However, ApaI and BsmI polymorphisms are not related with childhood asthma susceptibility.

## Materials and Methods

### Selection Strategy

The published studies were searched from numerous databases including Embase, PubMed, WANFANG data, China National Knowledge Infrastructure (CNKI), China Science and technology journal database (CQVIP), etc. The comprehensive systematic search process was exploited till 12 April, 2021 using the following key words: (“Vitamin D receptor” OR “VDR”) AND (“polymorphisms” OR “polymorphism” OR “variant” OR “mutant”) AND (“children” OR “child” OR “teenager” OR “pediatric”). The selection strategies in Pubmed and Embase were shown in Supplementary Tables 1, 2. Moreover, in order to enroll more researches, print-out literatures, reviews, and the references of included articles were also retrieved.

### Study Selection

The following inclusion criteria were designed: (1) the study was designed as a case-control study or cohort study; (2) the subjects in the experiment group were children and/or adolescents with asthma, and subjects in the control group were healthy children and/or adolescents; (3) The study explored the association of VDR ApaI (rs7975232), TaqI (rs731236), BsmI (rs1544410), FokI (rs2228570) gene polymorphisms and asthma susceptibility; (4) genotype data were reported or could be calculated based on information provided in the study.

When the control group and the case group were family members or close family members, the study would be excluded. The non-research articles, such as reviews, comments, and conference summaries, would be excluded. When duplicated studies were found or same data were showed in more than one study, the study with the most specific information would be included in the present study, and other duplicated articles would be excluded.

### Data Extraction and Quality Assessment

Based on the designed criteria, studies were screened by two investigators independently. According to the standardized form, the information including year of publication, the name of the first author, research regions, the demographic information (age, sample size, source of the control group), polymorphism detection methods, the ethnicity of the included population, and genotype data, etc.

Newcastle-Ottawa Scale (NOS) criteria was used to assess the methodological quality of included studies, and the scale was assessed according to three aspects including subjects selection, comparability, and exposure (14). The study with a score of five or more would be considered as moderate quality, and the study with a score of four or less would be considered as poor quality.

When data extraction was finished, the extraction form would be exchanged, and the disagreements were solved by discussing.

### Statistic Analysis

Firstly, the Hardy-Weinberg equilibrium test (HWE) of the frequency distribution of genotypes among controls was performed. We defined the population were not in the HWE if P < 0.05. For each single nucleotide polymorphisms (SNP), we examined four models, including computational additive model [m (mutation) vs. W (Wild)], co dominant model (mm vs. WW, Wm vs. WW), dominant model (mm+ Wm vs. WW), and recessive model (mm vs. WW + Wm). The effect of VDR polymorphisms in the childhood asthma susceptibility was assessed based on the pooled odds ratio (OR) and its 95% confidence interval (95%CI). Heterogeneity among individual studies was assessed using Cochran's Q test and I^2^ test (15). If *P* < 0.05, and/or I^2^>50%, suggesting obvious heterogeneity between the studies, the random effects model would be selected to calculate the pooled data; If *P* ≥ 0.05 and/or I^2^ ≤ 50%, the fixed effect model would be used. RevMan 5.3 software and Stata 11.0 were enrolled to perform all statistical analyses.

## Results

### Studies Inclusion

**Figure 1:**
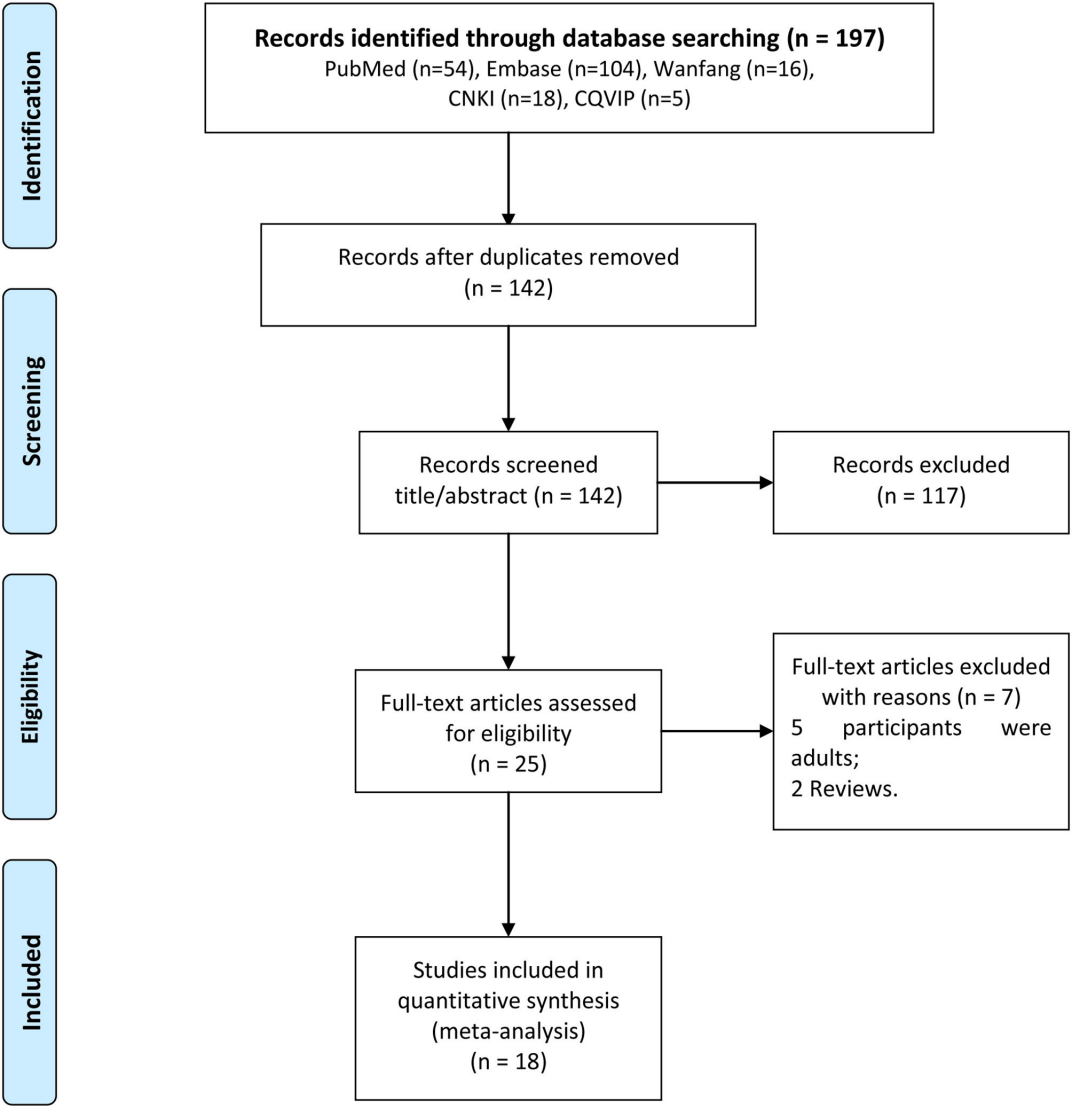
The detailed flow chart for study selection.

The detailed information associated with search process was shown in Figure 1. In this study, a total of 197 studies were firstly searched, including 54 articles in PubMed, 104 articles in Embase, 16 articles in Wanfang data, 18 articles in CNKI, and 5 articles in CQVIP. After removing 55 duplicated documents, there were 142 articles remaining. After that, we excluded 117 articles after browsing the titles and reading the abstract. Then, total 25 articles were fully reviewed, and seven articles were excluded, including five articles with adults as study subjects and two reviews. Finally, 18 articles were included in this meta-analysis (8–13, 16–27).

### The Baseline Characteristics and Quality Assessment of Included Studies

As shown in Table 1, total 3,495 subjects including 1,392 cases in asthma group and 2,103 cases in control group were enrolled in the present study. The studies included in the meta-analysis were all published from 2010 to 2020. Among these articles, the subjects in eight studies were Asians, eight articles were Caucasians, one study were Americans, and one study were African-Americans. For subjects in the control group, there were two studies with population-based controls and 16 studies with hospital-based controls.

**Table 1:** Characteristics of the included studies.

**Study**	**Area**	**Ethnicity**	**Source of control**	**Diagnostic of asthma**	**Polymorphisms**	**Group**	**n, M/F**	**Age, years**
Ahmed et al. (9)	Egypt	European	HB	GINA guidelines	ApaI, TaqI, BsmI	Asthma	50, 28/22	12.66 ± 3.34
						Control	50, 29/21	12.08 ± 3.53
Batmaz et al. (16)	Turkey	European	HB	GINA guidelines	ApaI, TaqI, FokI, BsmI	Asthma	30, 20/10	11.74 ± 2.4
						Control	30, 13/17	11.31 ± 2.27
Einisman et al. (17)	Chile	American	HB	GINA guidelines	ApaI, TaqI, FokI	Asthma	75, 43/32	9.1 (3.5) $
						Control	227, 114/113	10.3 (7.9)
Hou et al. (10)	China	Asia	HB	DPGPBA (2008)	ApaI, BsmI	Asthma	70, 43/27	8.84 ± 3.21
						Control	70, 37/33	8.04 ± 3.01
Hutchinson et al. (11)	Ireland	European	HB	GINA guidelines	ApaI, TaqI	Asthma	44, 23/21	8.7 (6–13, 15–17)
						Control	57, NR	NR
Iordanidou et al. (18)	Greece	European	HB	GINA guidelines	ApaI, TaqI, FokI, BsmI	Asthma	127, 82/45	8.4 ± 2.3
						Control	91, 41/50	9.5 ± 3.8
Ismail et al. (12)	Egypt	European	HB	GINA guidelines	FokI	Asthma	51, 28/23	8.6 ± 2.7
						Control	33, 18/15	7.8 ± 2.6
Kilic et al. (12)	Turkey	European	HB	GINA guidelines	ApaI, TaqI, FokI	Asthma	100, 52/48	9.5 ± 2.8
						Control	80, 42/38	9.5 ± 2.5
Liu et al. (20)	China	Asia	HB	DPGPBA (2008)	ApaI, TaqI, FokI, BsmI	Asthma	41, 24/17	3.9 ± 1.2
						Control	41, 23/18	3.7 ± 1.3
Ma et al. (21)	China	Asia	PB	DPGPBA (2008)	ApaI, TaqI, FokI, BsmI	Asthma	60, 32/28	10.2
						Control	60, 30/30	10.6
Maalmi et al. (22)	Tunisia	European	HB	DPGPBA (2008)	ApaI, TaqI, FokI, BsmI	Asthma	155, 59/96	9.1 (4–13, 15–17)
						Control	225, 99/126	9.5 (2–13, 15–17)
Mo et al. (23)	China	Asia	HB	DPGPBA (2008)	ApaI, BsmI	Asthma	71, NR	NR
						Control	71, NR	NR
Papadopoulou et al. (24)	Cyprus	European	PB	NR	ApaI, TaqI, BsmI	Asthma	69, 30/39	16.9 (15.9-18.1)
						Control	671, 282/389	17.0 (15.9-18.0)
Pillai et al. (25)	USA	African- American	HB	NAEPP (2007)	ApaI, TaqI, FokI	Asthma	139, 81/58	11.2 ± 3.5
						Control	74, 26/48	11.8 ± 4.3
Zhang et al. (26)	China	Asia	HB	NR	ApaI, FokI, BsmI	Asthma	143, 86/57	7.56 ± 2.39
						Control	143, 87/56	7.28 ± 2.54
Zhao et al. (27)	China	Asia	HB	DPGPBA (2008)	ApaI, TaqI, FokI, BsmI	Asthma	40, 22/18	3.41 ± 1.07
						Control	40, 21/19	3.37 ± 1.04
Zhen and Wang (8)	China	Asia	HB	NR	ApaI	Asthma	30, 17/13	5.70 ± 2.84
						Control	40, 22/18	5.53 ± 2.93
Zhu et al. (13)	China	Asia	HB	DPGPBA (2008)	FokI, BsmI	Asthma	97, 50/47	8.76 ± 1.22
						Control	100, 55/45	8.60 ± 1.16

*$, median (IQR); HB, hospital-based; PB, population-based; PCR, Polymerase chain reaction; RT-PCR, Realtime polymerase chain reaction; RFLP, restriction fragment lengths polymorphism; NR, not reported; BMI, body mass index; GINA, the Global Initiative for Asthma; NAEPP, National Asthma Education and Prevention Program (2007) (28)*.

The genotype data and HWE test results of the case group and the control group were shown in Table 2. NOS scores of all included studies ranged from 5 to 8, suggesting an overall moderate methodological quality (Table 3).

**Table 2:** Frequency distribution of gene polymorphisms in the experimental group and the control group.

**References**	**Country**	**Case group**				**Control group**				***P*_value_ for HWE**
		**N**	**WW**	**WM**	**MM**	**N**	**WW**	**WM**	**MM**	
**ApaI (rs7975232)**										
Ahmed et al. (9)	Egypt	50	25	15	10	50	20	20	10	0.2386
Batmaz et al. (16)	Turkey	30	5	25	0	30	7	23	0	0.0070
Einisman et al. (17)	Chile	70	21	35	14	50	10	28	12	0.3891
Hou et al. (10)	China	70	4	30	36	70	0	5	65	0.7567
Hutchinson et al. (11)	Ireland	44	11	23	10	57	5	20	32	0.4721
Iordanidou et al. (18)	Greece	127	41	63	23	91	35	41	15	0.6120
Kilic et al. (12)	Turkey	100	18	60	22	80	26	42	12	0.4569
Liu et al. (20)	China	41	7	15	19	41	1	4	36	0.1599
Ma et al. (21)	China	60	46	7	7	60	48	4	8	<0.0001
Maalmi et al. (22)	Tunisia	155	92	57	6	225	142	70	13	0.2729
Mo et al. (23)	China	71	4	31	36	71	14	28	29	0.1416
Papadopoulou et al. (24)	Cyprus	61	19	34	8	633	232	312	89	0.3290
Pillai et al. (25)	USA	125	55	59	11	72	35	33	4	0.2762
Zhang et al. (26)	China	143	54	75	14	143	8	69	66	0.0637
Zhao et al. (27)	China	40	0	15	25	40	0	27	13	0.0013
Zhen and Wang (8)	China	30	3	2	25	40	6	14	20	0.2061
**TaqI (rs731236)**										
Ahmed et al. (9)	Egypt	50	5	30	15	50	10	40	0	<0.0001
Batmaz et al. (16)	Turkey	30	18	9	3	30	15	10	5	0.1709
Einisman et al. (17)	Chile	72	35	34	3	50	24	24	2	0.1780
Hutchinson et al. (11)	Ireland	44	17	21	6	57	34	23	0	0.0564
Iordanidou et al. (18)	Greece	127	43	68	16	91	35	38	18	0.1990
Kilic et al. (12)	Turkey	100	31	61	8	80	28	32	20	0.0861
Liu et al. (20)	China	41	6	11	24	41	1	5	35	0.1607
Ma et al. (21)	China	60	6	7	47	60	5	6	49	<0.0001
Maalmi et al. (22)	Tunisia	155	59	81	15	225	79	101	45	0.2230
Papadopoulou et al. (24)	Cyprus	61	28	20	13	630	224	325	81	0.0276
Pillai et al. (25)	USA	118	52	55	11	74	40	31	3	0.3137
Zhao et al. (27)	China	40	26	14	0	40	13	27	0	0.0013
**BsmI (rs1544410)**										
Ahmed et al. (9)	Egypt	50	10	25	15	50	20	20	10	0.2386
Batmaz et al. (16)	Turkey	30	2	12	16	30	5	13	12	0.6477
Hou et al. (10)	China	70	0	4	66	70	0	5	65	0.7567
Iordanidou et al. (18)	Greece	127	20	67	40	91	19	39	33	0.2442
Liu et al. (20)	China	41	9	11	21	41	1	4	36	0.0723
Ma et al. (21)	China	60	5	10	45	60	6	6	48	<0.0001
Maalmi et al. (22)	Tunisia	155	34	72	49	225	26	119	80	0.0663
Mo et al. (23)	China	71	0	5	66	71	0	4	67	0.8070
Papadopoulou et al. (24)	Cyprus	63	11	32	20	631	127	327	177	0.2801
Zhang et al. (26)	China	143	56	74	13	143	72	65	6	0.0635
Zhao et al. (27)	China	40	0	23	17	40	0	11	29	0.3133
Zhu et al. (13)	China	97	0	10	87	100	0	2	98	0.9195
**FokI (rs2228570)**										
Batmaz et al. (16)	Turkey	30	19	11	0	30	12	12	6	0.3613
Einisman et al. (17)	Chile	73	11	62	0	50	5	45	0	<0.0001
Iordanidou et al. (18)	Greece	127	67	54	6	91	38	45	8	0.2958
Ismail et al. (19)	Egypt	51	29	22	0	33	12	14	7	0.4497
Kilic et al. (12)	Turkey	100	58	33	9	80	48	28	4	0.9744
Liu et al. (20)	China	41	8	13	20	41	1	5	35	0.1607
Ma et al. (21)	China	60	12	24	24	60	18	31	11	0.7124
Maalmi et al. (22)	Tunisia	155	88	56	11	152	70	59	23	0.0808
Pillai et al. (25)	USA	122	76	41	5	74	42	29	3	0.4636
Zhang et al. (26)	China	143	2	49	92	143	6	63	74	0.0975
Zhao et al. (27)	China	40	19	14	7	40	15	16	9	0.2508
Zhu et al. (13)	China	97	27	48	22	100	30	45	25	0.3283

**Table 3:** Quality assessment of the included studies.

**References**	**Representativeness of the cases**	**Case definition adequate**	**Ascertainment of exposure**	**Same method of ascertainment for cases and controls**	**Control for important factor or additional factor**	**Selection of controls**	**Definition of controls**	**Non-response rate**	**Total quality scores**
Ahmed et al. (9)	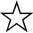	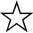	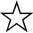	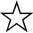	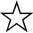	–	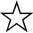	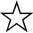	7
Batmaz et al. (16)	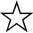	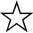	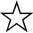	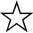	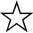	–	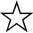	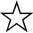	7
Einisman et al. (17)	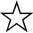	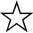	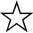	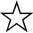	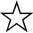	–	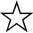	–	6
Hou et al. (10)	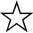	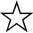	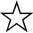	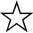	–	–	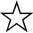	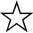	6
Hutchinson et al. (11)	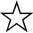	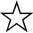	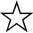	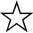	–	–	–	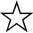	5
Iordanidou et al. (18)	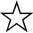	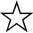	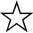	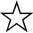	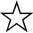	–	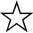	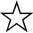	7
Ismail et al. (19)	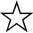	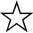	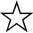	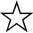	–	–	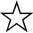	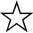	6
Kilic et al. (12)	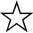	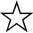	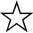	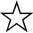	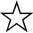	–	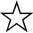	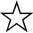	7
Liu et al. (20)	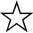	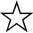	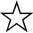	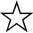	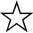	–	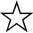	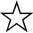	7
Ma et al. (21)	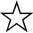	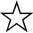	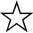	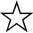	–	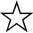	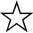	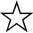	6
Maalmi et al. (22)	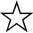	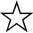	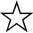	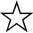	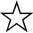	–	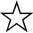	–	6
Mo et al. (23)	–	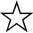	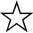	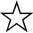	–	–	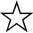	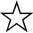	5
Papadopoulou et al. (24)	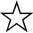	–	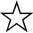	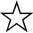	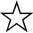	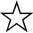	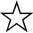	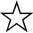	7
Pillai et al. (25)	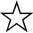	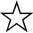	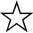	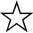	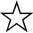	–	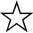	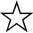	7
Zhang et al. (26)	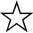	–	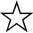	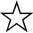	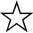	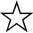	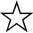	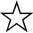	7
Zhao et al. (27)	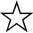	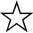	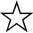	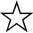	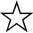	–	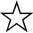	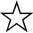	7
Zhen and Wang (8)	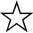	–	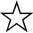	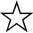	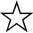	–	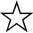	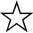	6
Zhu et al. (13)	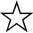	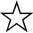	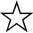	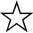	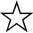	–	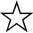	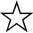	7

### Meta-Analysis of VDR Polymorphism and Asthma

#### ApaI (rs7975232)

**Figure 2:**
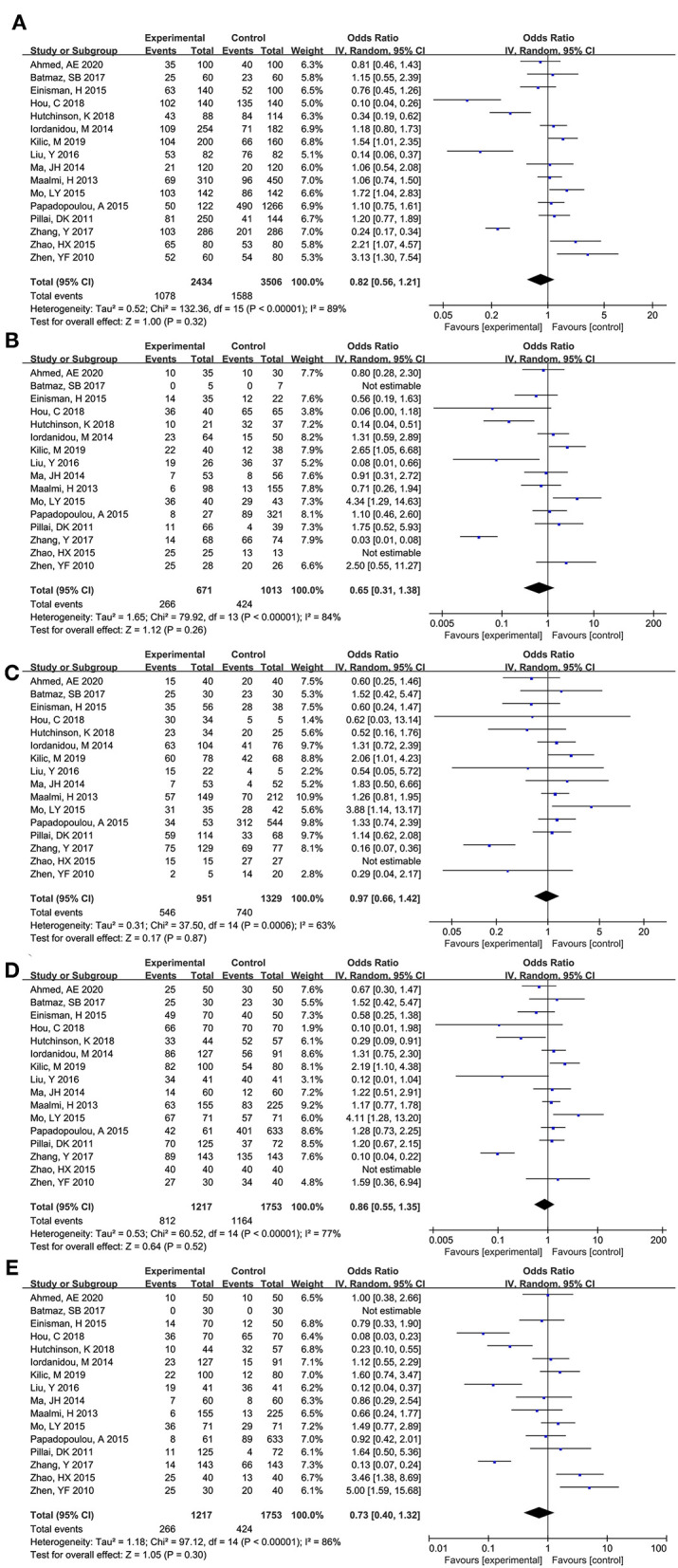
Forest plot for meta-analyzing the association between Vitamin D receptor ApaI (rs7975232) polymorphisms and childhood asthma. **(A)** additive model: a vs. A; **(B)** co dominant model: aa vs. AA; **(C)** co dominant model: Aa vs. AA; **(D)** dominant model: aa+Aa vs. AA; **(E)** recessive model: aa vs. AA+Aa.

As shown in Figure 2, total 16 articles reported the association between ApaI (rs7975232) and asthma risk (8–12, 16–18, 20–27). Obvious heterogeneity across studies was observed in additive model (a vs. A: I^2^ = 89%, *P* < 0.00001), co dominant model (aa vs. AA: I^2^ = 84%, *P* < 0.00001; Aa vs. AA: I^2^ = 63%, *P* < 0.0006), dominant model (aa+Aa vs. AA: I^2^ = 77%, *P* < 0.00001), and recessive model (aa vs. AA+Aa: I^2^ = 86%, *P* < 0.00001). No significant association between ApaI (rs7975232) and asthma risk was calculated across additive model (a vs. A: OR (95%CI) = 0.82 (0.56, 1.21), *P* = 0.317), co dominant model (aa vs. AA: OR (95%CI) = 0.65 (0.31, 1.38), *P* = 0.263; Aa vs. AA: OR (95%CI) = 0.97 (0.66, 1.42), *P* = 0.866), dominant model (aa+Aa vs. AA: OR (95%CI) = 0.86 (0.55, 1.35), *P* = 0.520), and recessive model (aa vs. AA+Aa: OR (95%CI) = 0.73 (0.40, 1.32), *P* = 0.295).

The subgroup analysis was performed stratified by ethnicity, HWE, and source of controls (Table 4). No significant association was observed in all subgroup analysis (P>0.05). Meanwhile, the results of heterogeneity analysis showed that ethnicity, HWE, and source of subjects were not the source of heterogeneity.

**Table 4:** Outcomes of the subgroup analysis.

**Model**	**No. of studies**	**Heterogeneity test**		**Effect size**	
		**I^**2**^ (%)**	**P_**H**_**	**OR (95% CI)**	***P* value**
**ApaI (rs7975232)**					
a vs. A	16	88.7	<0.001	0.82 (0.56, 1.21)	0.317
Ethnicity					
American	1	NA	NA	0.76 (0.45, 1.26)	0.285
Asian	7	93.6	<0.001	0.65 (0.25, 1.70)	0.378
Caucasians	7	67.3	0.005	0.98 (0.72, 1.33)	0.876
African-American	1	NA	NA	1.20 (0.77, 1.89)	0.417
HWE					
Yes	13	90.3	<0.001	0.73 (0.47, 1.13)	0.163
No	3	17.7	0.297	1.37 (0.88, 2.16)	0.167
Source					
HB	14	90.0	<0.001	0.79 (0.50, 1.22)	0.287
PB	2	0	0.927	1.09 (0.78, 1.52)	0.607
aa vs. AA	16	83.7	<0.001	0.65 (0.31, 1.38)	0.263
Ethnicity					
American	1	NA	NA	0.56 (0.19, 1.63)	0.285
Asian	7	90.8	<0.001	0.37 (0.06, 2.48)	0.305
Caucasians	7	65.2	0.013	0.89 (0.46, 1.74)	0.737
African-American	1	NA	NA	1.75 (0.52, 5.93)	0.369
HWE					
Yes	13	85.0	<0.001	0.63 (0.28, 1.42)	0.264
No	3	NA	NA	0.91 (0.31, 2.72)	0.280
Source					
HB	14	86.0	<0.001	0.59 (0.24, 1.46)	0.254
PB	2	0	0.795	1.02 (0.52, 2.01)	0.948
Aa vs. AA	16	62.7	<0.001	0.97 (0.66, 1.42)	0.866
Ethnicity					
American	1	NA	NA	0.60 (0.24, 1.47)	0.260
Asian	7	72.9	<0.001	0.71 (0.19, 2.67)	0.615
Caucasians	7	9.2	0.358	1.24 (0.94, 1.63)	0.128
African-American	1	NA	NA	1.14 (0.62, 2.08)	0.674
HWE					
Yes	13	67.0	<0.001	0.90 (0.59, 1.38)	0.637
No	3	0	0.844	1.67 (0.67, 4.14)	0.272
Source					
HB	14	66.6	<0.001	0.89 (0.57, 1.39)	0.612
PB	2	0.0	0.662	1.40 (0.82, 2.40)	0.213
aa+Aa vs. AA	16	76.9	<0.001	0.86 (0.55, 1.35)	0.520
Ethnicity					
American	1	NA	NA	0.58 (0.25, 1.38)	0.220
Asian	7	86.9	<0.001	0.53 (0.12, 2.25)	0.391
Caucasians	7	46.8	0.080	1.14 (0.79, 1.62)	0.488
African-American	1	NA	NA	1.20 (0.67, 2.15)	0.532
HWE					
Yes	13	79.9	<0.001	0.80 (0.48, 1.33)	0.393
No	3	0	0.778	1.31 (0.64, 2.68)	0.467
Source					
HB	14	79.8	<0.001	0.79 (0.47, 1.36)	0.400
PB	2	0	0.926	1.26 (0.78, 2.03)	0.339
aa vs. AA+Aa	16	85.6	<0.001	0.73 (0.40, 1.32)	0.295
Ethnicity					
American	1	NA	NA	0.79 (0.33, 1.90)	0.600
Asian	7	92.5	<0.001	0.60 (0.17, 2.04)	0.409
Caucasians	7	58.5	0.034	0.81 (0.48, 1.39)	0.446
African-American	1	NA	NA	1.64 (0.50, 5.36)	0.412
HWE					
Yes	13	85.8	<0.001	0.64 (0.34, 1.20)	0.165
No	3	72.9	0.055	1.78 (0.45, 6.96)	0.409
Source					
HB	14	87.6	<0.001	0.70 (0.35, 1.40)	0.319
PB	2	0.0	0.916	0.90 (0.48, 1.69)	0.745
**TaqI (rs731236)**					
t vs. T	12	71.1	<0.001	0.93 (0.70, 1.23)	0.608
Ethnicity					
American	1	NA	NA	0.99 (0.56, 1.75)	0.970
Asian	3	55.4	0.106	0.45 (0.23, 0.89)	0.022
Caucasians	7	71.5	0.002	1.06 (0.77, 1.47)	0.711
African-American	1	NA	NA	1.45 (0.92, 2.30)	0.112
HWE					
Yes	8	70.8	0.001	0.91 (0.66, 1.27)	0.582
No	4	77.9	0.004	0.96 (0.52, 1.78)	0.888
Source					
HB	10	76.3	<0.001	0.93 (0.66, 1.30)	0.670
PB	2	0	0.699	0.93 (0.66, 1.30)	0.661
tt vs. TT	12	63.5	0.002	0.92 (0.49, 1.70)	0.783
Ethnicity					
American	1	NA	NA	1.03 (0.16, 6.63)	0.976
Asian	3	56.5	0.129	0.37 (0.06, 2.40)	0.300
Caucasians	7	71.0	0.002	0.96 (0.44, 2.09)	0.912
African-American	1	NA	NA	2.82 (0.74, 10.79)	0.130
HWE					
Yes	8	56.2	0.025	0.71 (0.36, 1.40)	0.320
No	4	70.6	0.033	2.01 (0.46, 8.77)	0.351
Source					
HB	10	67.7	0.002	0.93 (0.42, 2.07)	0.857
PB	2	3.5	0.407	1.15 (0.62, 2.12)	0.665
Tt vs. TT	12	50.2	0.024	1.00 (0.72, 1.38)	0.996
Ethnicity					
American	1	NA	NA	0.97 (0.46, 2.03)	0.939
Asian	3	0	0.379	0.36 (0.17, 0.77)	0.009
Caucasians	7	48.6	0.070	1.13 (0.78, 1.65)	0.513
African-American	1	NA	NA	1.36 (0.75, 2.49)	0.312
HWE					
Yes	8	0	0.693	1.26 (0.99, 1.60)	0.064
No	4	49.2	0.116	0.57 (0.29, 1.15)	0.120
Source					
HB	10	41.4	0.082	1.11 (0.81, 1.53)	0.521
PB	2	0.0	0.438	0.53 (0.31, 0.94)	0.029
tt+Tt vs. TT	12	53.3	0.015	0.96 (0.70, 1.32)	0.817
Ethnicity					
American	1	NA	NA	0.98 (0.47, 2.01)	0.947
Asian	3	29.0	0.244	0.36 (0.15, 0.87)	0.024
Caucasians	7	42.7	0.106	1.08 (0.77, 1.50)	0.663
African-American	1	NA	NA	1.49 (0.83, 2.68)	0.178
HWE					
Yes	8	30.6	0.184	1.12 (0.84, 1.50)	0.437
No	4	64.1	0.039	0.70 (0.32, 1.51)	0.362
Source					
HB	10	56.6	0.014	1.02 (0.71, 1.47)	0.901
PB	2	0	0.739	0.67 (0.41, 1.10)	0.112
tt vs. TT+Tt	12	72.6	<0.001	0.87 (0.47, 1.62)	0.658
Ethnicity					
American	1	NA	NA	1.04 (0.17, 6.48)	0.964
Asian	3	65.5	0.089	0.46 (0.14, 1.50)	0.198
Caucasians	7	79.3	<0.001	0.97 (0.41, 2.28)	0.941
African-American	1	NA	NA	2.43 (0.66, 9.03)	0.184
HWE					
Yes	8	57.8	0.020	0.60 (0.32, 1.09)	0.095
No	4	73.5	0.023	2.06 (0.59, 7.27)	0.259
Source					
HB	10	69.2	0.001	0.78 (0.38, 1.61)	0.503
PB	2	51.7	0.150	1.30 (0.59, 2.86)	0.521
**BsmI (rs1544410)**					
b vs. B	12	73.2	<0.001	0.87 (0.62, 1.21)	0.408
Ethnicity					
Asian	7	79.9	<0.001	0.57 (0.28, 1.17)	0.128
Caucasians	5	62.5	0.031	1.12 (0.82, 1.54)	0.481
HWE					
Yes	11	75.6	<0.001	0.86 (0.60, 1.23)	0.419
No	1	NA	NA	0.88 (0.44, 1.77)	0.724
Source					
HB	10	77.6	<0.001	0.81 (0.54, 1.24)	0.336
PB	2	0	0.527	1.08 (0.78, 1.49)	0.662
bb vs. BB	12	68.0	0.003	1.16 (0.61, 2.21)	0.665
Ethnicity					
Asian	7	79.5	0.008	0.74 (0.12, 4.40)	0.741
Caucasians	5	66.2	0.019	1.24 (0.62, 2.50)	0.539
HWE					
Yes	11	72.6	0.001	1.16 (0.56, 2.40)	0.690
No	1	NA	NA	1.13 (0.32, 3.94)	0.854
Source					
HB	10	76.7	0.001	1.12 (0.45, 2.76)	0.805
PB	2	0	0.844	1.25 (0.65, 2.42)	0.501
Bb vs. BB	12	55.6	0.027	1.22 (0.76, 1.96)	0.409
Ethnicity					
Asian	7	0	0.401	1.42 (0.90, 2.23)	0.130
Caucasians	5	68.4	0.013	1.22 (0.62, 2.40)	0.561
HWE					
Yes	11	60.8	0.018	1.18 (0.71, 1.96)	0.524
No	1	NA	NA	2.00 (0.42, 9.52)	0.384
Source					
HB	10	67.3	0.009	1.20 (0.64, 2.24)	0.576
PB	2	0.0	0.514	1.25 (0.65, 2.39)	0.505
bb+Bb vs. BB	12	68.5	0.002	1.14 (0.67, 1.93)	0.627
Ethnicity					
Asian	7	70.5	0.034	0.80 (0.23, 2.81)	0.732
Caucasians	5	72.2	0.006	1.25 (0.64, 2.47)	0.515
HWE					
Yes	11	73.0	0.001	1.13 (0.63, 2.01)	0.686
No	1	NA	NA	1.22 (0.35, 4.24)	0.752
Source					
HB	10	77.4	<0.001	1.10 (0.54, 2.25)	0.788
PB	2	0	0.972	1.20 (0.66, 2.17)	0.552
bb vs. BB+Bb	12	61.5	0.003	0.80 (0.54, 1.20)	0.278
Ethnicity					
Asian	7	69.2	0.003	0.55 (0.25, 1.18)	0.124
Caucasians	5	0	0.414	1.01 (0.77, 1.32)	0.948
HWE					
Yes	11	64.9	0.001	0.80 (0.52, 1.24)	0.326
No	1	NA	NA	0.75 (0.32, 1.77)	0.513
Source					
HB	10	66.5	0.001	0.75 (0.46, 1.24)	0.267
PB	2	0.0	0.375	1.04 (0.65, 1.66)	0.870
**FokI (rs2228570)**					
f vs. F	12	76.7	<0.001	0.78 (0.57, 1.05)	0.102
Ethnicity					
American	1	NA	NA	0.90 (0.54, 1.51)	0.694
Asian	5	84.4	<0.001	0.90 (0.50, 1.63)	0.721
Caucasians	5	62.7	0.030	0.63 (0.43, 0.92)	0.015
African-American	1	NA	NA	0.85 (0.52, 1.39)	0.524
HWE					
Yes	11	78.8	<0.001	0.76 (0.55, 1.06)	0.107
No	1	NA	NA	0.90 (0.54, 1.51)	0.694
Source					
HB	11	73.2	<0.001	0.72 (0.54, 0.97)	0.030
PB	1	NA	NA	1.90 (1.14, 3.17)	0.015
ff vs. FF	12	67.6	0.001	0.67 (0.34, 1.34)	0.260
Ethnicity					
American	1	NA	NA	NA	NA
Asian	5	70.7	0.008	1.05 (0.38, 2.91)	0.925
Caucasians	5	62.9	0.029	0.37 (0.13, 1.07)	0.067
African-American	1	NA	NA	0.92 (0.21, 4.05)	0.913
HWE					
Yes	11	67.6	0.001	0.67 (0.34, 1.34)	0.260
No	1	NA	NA	NA	NA
Source					
HB	11	59.3	0.008	0.57 (0.29, 1.11)	0.095
PB	1	NA	NA	3.27 (1.18, 9.09)	0.023
Ff vs. FF	12	0	0.890	0.82 (0.65, 1.02)	0.071
Ethnicity					
American	1	NA	NA	0.63 (0.20, 1.93)	0.415
Asian	5	72.9	<0.001	1.06 (0.68, 1.65)	0.796
Caucasians	5	32.1	0.172	0.75 (0.56, 1.00)	0.052
African-American	1	NA	NA	0.78 (0.43, 1.43)	0.425
HWE					
Yes	11	0	0.854	0.82 (0.66, 1.03)	0.093
No	1	NA	NA	0.63 (0.20, 1.93)	0.415
Source					
HB	11	0	0.883	0.80 (0.64, 1.00)	0.052
PB	1	NA	NA	1.16 (0.47, 2.87)	0.746
ff+Ff vs. FF	12	34.7	0.112	0.77 (0.63, 0.95)	0.016
Ethnicity					
American	1	NA	NA	0.63 (0.20, 1.93)	0.415
Asian	5	53.6	0.071	1.08 (0.72, 1.63)	0.714
Caucasians	5	11.6	0.340	0.67 (0.51, 0.88)	0.004
African-American	1	NA	NA	0.79 (0.44, 1.43)	0.443
HWE					
Yes	11	40.2	0.081	0.78 (0.63, 0.96)	0.021
No	1	NA	NA	0.63 (0.20, 1.93)	0.415
Source					
HB	11	24.1	0.214	0.73 (0.59, 0.91)	0.005
PB	1	NA	NA	1.71 (0.74, 3.97)	0.208
ff vs. FF+Ff	12	74.2	<0.001	0.71 (0.39, 1.29)	0.266
Ethnicity					
American	1	NA	NA	NA	NA
Asian	5	81.6	<0.001	0.94 (0.42, 2.10)	0.880
Caucasians	5	59.8	0.041	0.43 (0.16, 1.17)	0.099
African-American	1	NA	NA	1.01 (0.23, 4.36)	0.988
HWE					
Yes	11	74.2	<0.001	0.71 (0.39, 1.29)	0.266
No	1	NA	NA	NA	NA
Source					
HB	11	70.7	<0.001	0.60 (0.33, 1.11)	0.103
PB	1	NA	NA	2.97 (1.29, 6.83)	0.010

#### TaqI (rs731236)

**Figure 3:**
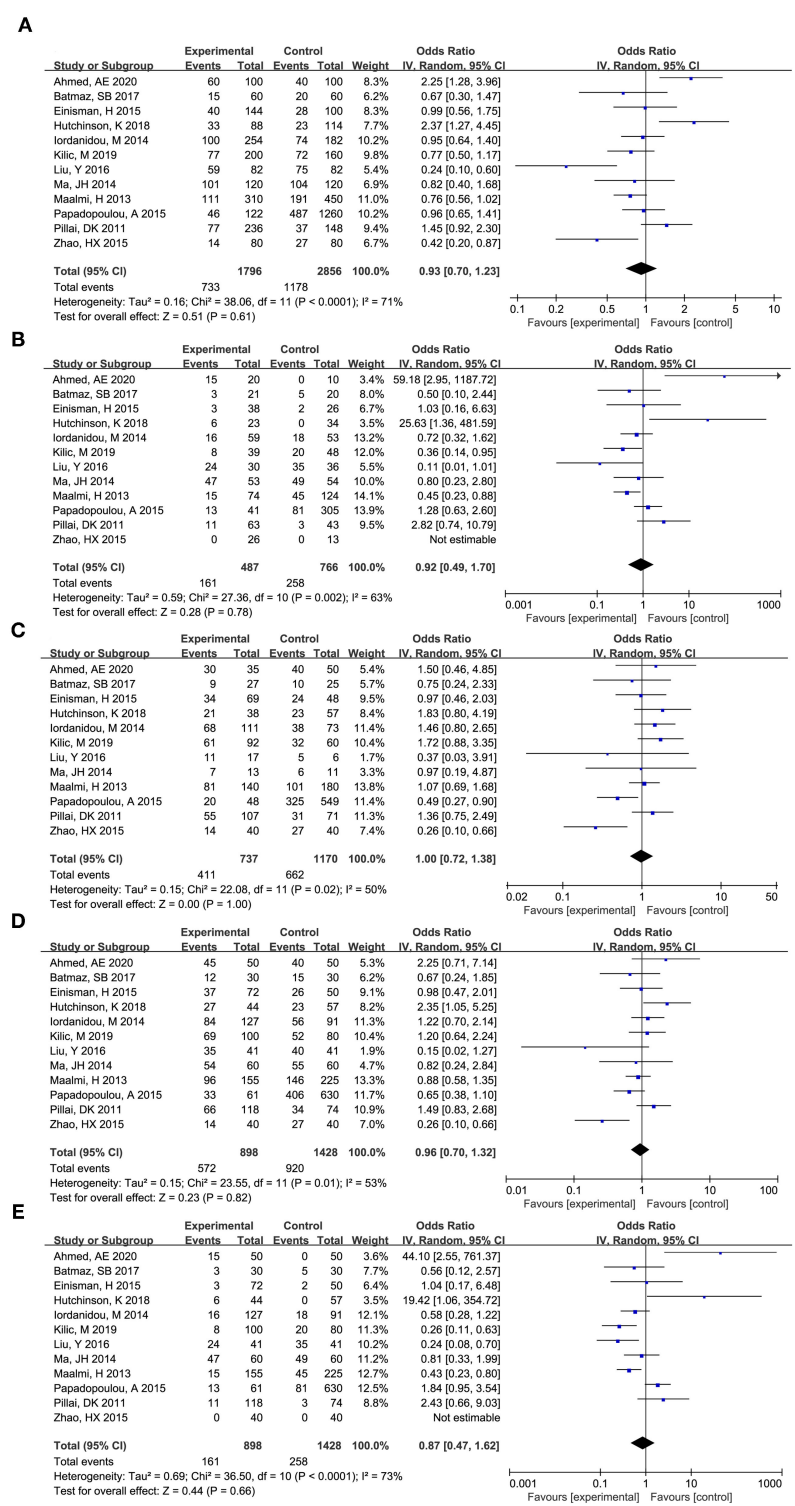
Forest plot for meta-analyzing the association between Vitamin D receptor TaqI (rs731236) polymorphisms and childhood asthma. **(A)** additive model: t vs. T; **(B)** co dominant model: tt vs. TT; **(C)** co dominant model: Tt vs. TT; **(D)** dominant model: tt+Tt vs. TT; **(E)** recessive model: tt vs. TT+Tt.

As shown in Figure 3, total 12 articles researched on the role of TaqI (rs731236) in asthma risk (9, 11, 12, 16–18, 20–25, 27). Obvious heterogeneity across studies was observed in additive model (t vs. T: I^2^ = 71%, *P* < 0.0001), co dominant model (tt vs. TT: I^2^ = 63%, *P* = 0.002; Tt vs. TT: I^2^ = 50%, *P* = 0.02), dominant model (tt+Tt vs. TT: I^2^ = 53%, *P* = 0.82), and recessive model (tt vs. TT+Tt: I^2^ = 73%, *P* < 0.0001). Thus, the randomed effects model was used to calculated the pooled data, and the results showed that no significant association between TaqI (rs731236) and asthma risk was observed across additive model (t vs. T: OR (95%CI) = 0.93 (0.70, 1.23), *P* = 0.608), co dominant model [tt vs. TT: OR (95%CI) = 0.92 (0.49, 1.70), *P* = 0.783; Tt vs. TT: OR (95%CI) = 1.00 (0.72, 1.38), *P* = 0.996], dominant model [tt+Tt vs. TT: OR (95%CI) = 0.96 (0.70, 1.32), *P* = 0.817], and recessive model [tt vs. TT+Tt: OR (95%CI) = 0.87 (0.47, 1.62), *P* = 0.658].

Further subgroup analysis showed that HWE and source of control were two sources for the obvious heterogeneity across co dominant model (Tt vs. TT). Notably, significant association was found in additive model [t vs. T: OR (95%CI) = 0.45 (0.23, 0.89), *P* = 0.022], co dominant model [Tt vs. TT: OR (95%CI) = 0.36 (0.17, 0.77), *P* = 0.009], and dominant model [tt+Tt vs. TT: OR (95%CI) = 0.36 (0.15, 0.87), *P* = 0.024] among Asians. Moreover, significant association was also found in the population-based subgroup in co dominant model [Tt vs. TT: OR (95%CI) = 0.53 (0.31, 0.94), *P* = 0.029].

#### BsmI (rs1544410)

**Figure 4:**
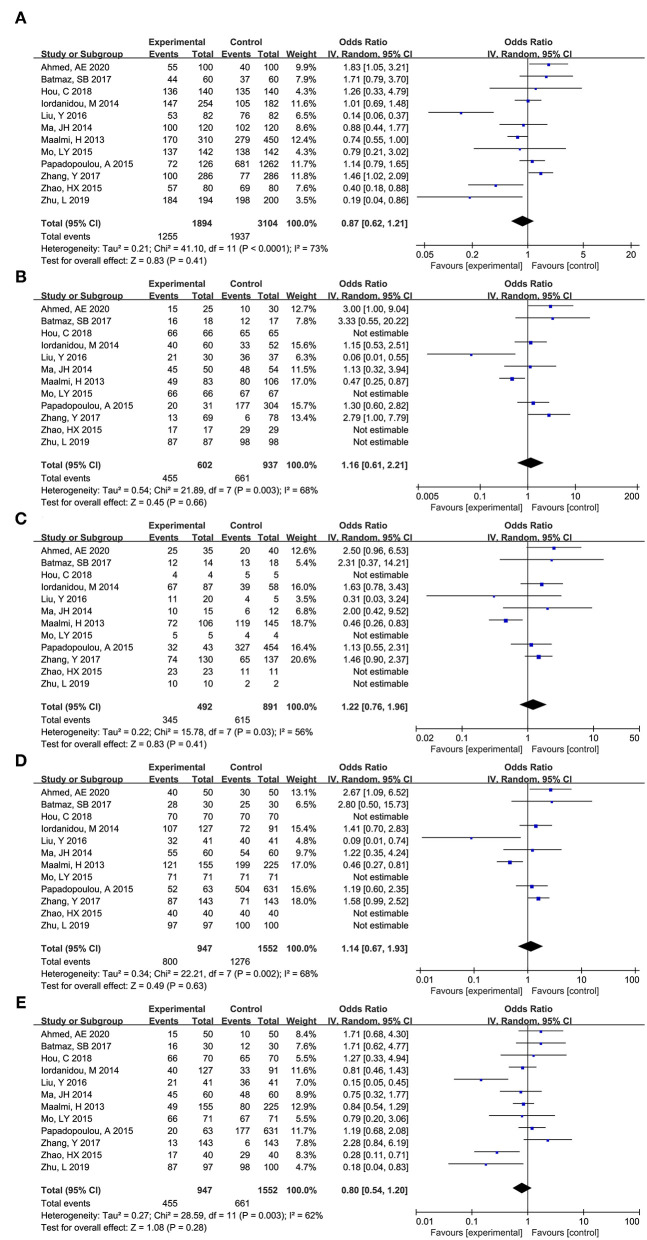
Forest plot for meta-analyzing the association between Vitamin D receptor BsmI (rs1544410) polymorphisms and childhood asthma. **(A)** additive model: b vs. B; **(B)** co dominant model: bb vs. BB; **(C)** co dominant model: Bb vs. BB; **(D)** dominant model: bb+Bb vs. BB; **(E)**: recessive model: bb vs. BB+Bb.

As shown in Figure 4, total 12 articles researched on the role of BsmI (rs1544410) in asthma risk (9, 10, 13, 16, 18, 20–24, 26, 27). Obvious heterogeneity across studies was observed in additive model (b vs. B: I^2^ = 73%, *P* < 0.0001), co dominant model (bb vs. BB: I^2^ = 68%, *P* = 0.003; Bb vs. BB: I^2^ = 56%, *P* = 0.03), dominant model (bb+Bb vs. BB: I^2^ = 68%, *P* = 0.002), and recessive model (bb vs. BB+Bb: I^2^ = 62%, *P* = 0.003). Thus, the randomed effects model was used to calculated the pooled data, and the results showed that no significant association between BsmI (rs1544410) and asthma risk was observed across additive model (b vs. B: OR (95%CI) = 0.87 (0.62, 1.21), *P* = 0.408), co dominant model (bb vs. BB: OR (95%CI) = 1.16 (0.61, 2.21), *P* = 0.665; Bb vs. BB: OR (95%CI) = 1.22 (0.76, 1.96), *P* = 0.409), dominant model (bb+Bb vs. BB: OR (95%CI) = 1.14 (0.67, 1.93), *P* = 0.627), and recessive model (bb vs. BB+Bb: OR (95%CI) = 0.80 (0.54, 1.20), *P* = 0.278).

Further subgroup analysis showed that no significant association was observed in all subgroup analysis (*P* > 0.05). Meanwhile, the results of heterogeneity analysis showed that ethnicity, HWE, and source of subjects were not the source of heterogeneity.

#### FokI (rs2228570)

**Figure 5:**
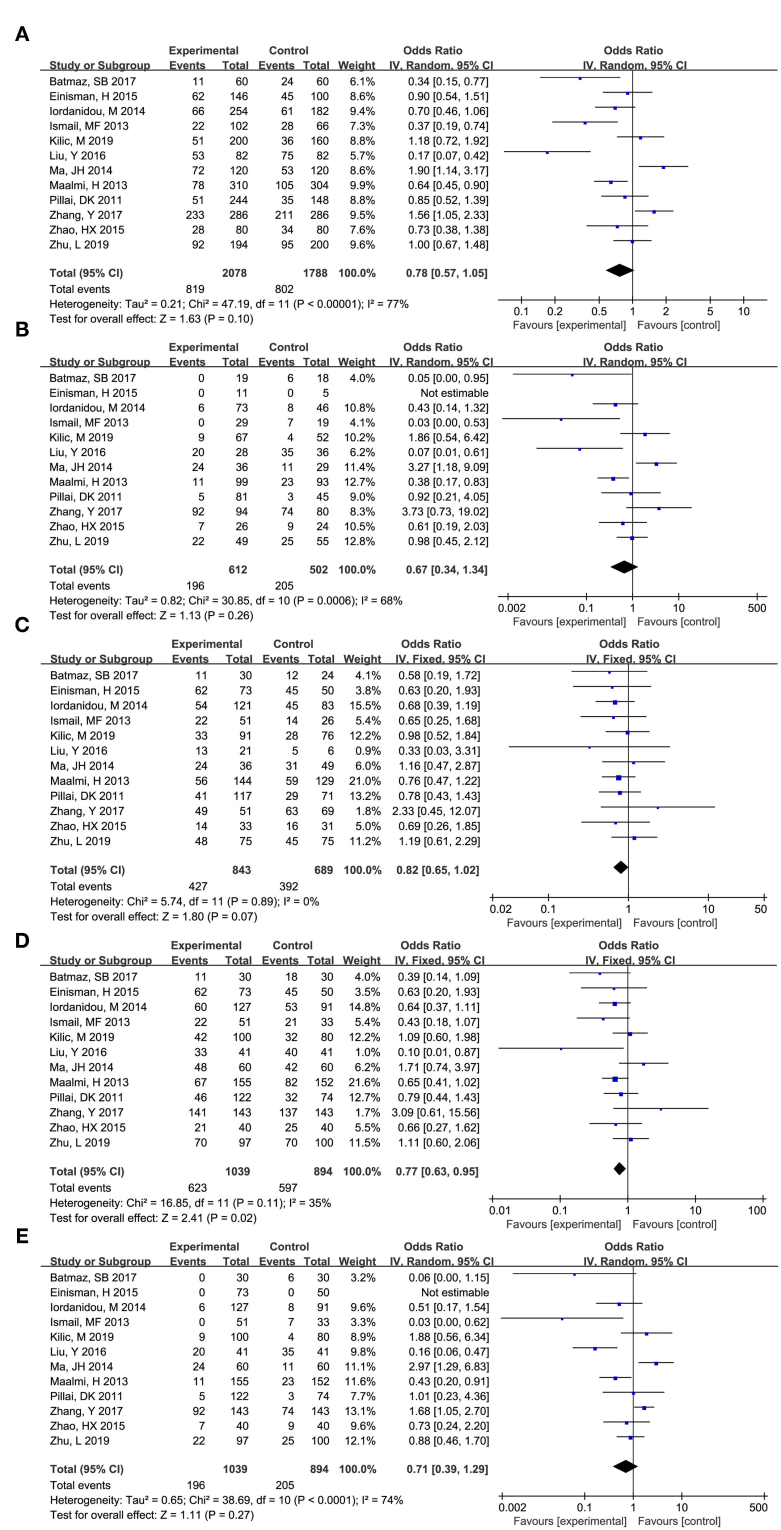
Forest plot for meta-analyzing the association between Vitamin D receptor FokI (rs2228570) polymorphisms and childhood asthma. **(A)** additive model: f vs. F; **(B)** co dominant model: ff vs. FF; **(C)** co dominant model: Ff vs. FF; **(D)** dominant model: ff+Ff vs. FF; **(E)**: recessive model: ff vs. FF+Ff.

As shown in Figure 5, total 12 articles researched on the role of FokI (rs2228570) in asthma risk (12, 13, 16–22, 25–27). Obvious heterogeneity across studies was observed in additive model (f vs. F: I^2^ = 77%, *P* < 0.00001), co dominant model (ff vs. FF: I^2^ = 68%, *P* = 0.0006), and recessive model (ff vs. FF+Ff: I^2^ = 74%, *P* < 0.0001). Thus, the randomed effects model was used to calculated the pooled data, and the results showed that no significant association between FokI (rs2228570) and asthma risk was observed across additive model (f vs. F: OR (95%CI) = 0.78 (0.57, 1.05), *P* = 0.102), co dominant model (ff vs. FF: OR (95%CI) = 0.67 (0.34, 1.34), *P* = 0.260), and recessive model (ff vs. FF+Ff: OR (95%CI) = 0.71 (0.39, 1.29), *P* = 0.266).

No significant obvious heterogeneity across studies was observed in co dominant model (Ff vs. FF: I^2^=0%, *P* = 0.89) and dominant model (ff+Ff vs. FF: I^2^=35%, *P* = 0.02), thus, the fixed effect model was used to calculate the pooled data, and the results showed that FokI (rs2228570) could significantly affect the risk of asthma across co dominant model (Ff vs. FF: OR (95%CI) = 0.82 (0.65, 1.02), *P* = 0.071) and dominant model (ff+Ff vs. FF: OR (95%CI) = 0.77 (0.63, 0.95), *P* = 0.016).

As for FokI (rs2228570), race, source of controls, HWE were not the source for the obvious heterogeneity. Subgroup analysis showed that FokI (rs2228570) SNP was significantly related with the risk of asthma in additive model (f vs. F: OR (95%CI) = 0.63 (0.43, 0.92), *P* = 0.015) and dominant model (ff+Ff vs. FF: OR (95%CI) = 0.67 (0.51, 0.88), *P* = 0.004) among Caucasians. Meanwhile, significant association was found in additive model (f vs. F: OR (95%CI) = 0.72 (0.54, 0.97), *P* = 0.03) and dominant model (ff+Ff vs. FF: OR (95%CI) = 0.67 (0.51, 0.88), *P* = 0.004) in the hospital-based subgroup. Significant association was found in additive model (f vs. F: OR (95%CI) = 1.90 (1.14, 3.17), *P* = 0.015), co dominant model (ff vs. FF: OR (95%CI) = 3.27 (1.18, 9.09), *P* = 0.023), and recessive model (ff vs. FF+Ff: OR (95%CI) = 2.97 (1.29, 6.83), *P* = 0.01) in population-based subgroup.

#### Publication Bias

No significant publication bias was observed for ApaI (rs7975232), TaqI (rs731236), BsmI (rs1544410), FokI (rs2228570) across the genotype models (P>0.05).

## Discussion

Among childhood, asthma is accepted as the most common chronic disease. Recently, accumulating evidence researched the function role of VDR gene polymorphism in childhood asthma, and four SNPs, including BsmI (rs1544410), ApaI (rs7975232), FokI (rs2228570), and TaqI (rs731236), were the main gene locuses (3, 29). Based on the meta-analysis, our data showed that FokI (rs2228570) could significantly affect the risk of childhood asthma across co dominant model and dominant model, especially among Caucasians. Notably, among Asians, significant correction between TaqI (rs731236) and childhood asthma was also found in additive model (t vs. T), co dominant model (Tt vs. TT), and dominant model (tt+Tt vs. TT), as well as population-based subgroup in co dominant model (Tt vs. TT). No relationship was found between childhood asthma and the polymorphisms of ApaI (rs7975232) and BsmI (rs1544410).

Previous evidence showed that the level of Vitamin D was closely related with airway remodeling, the number of T regulatory cells, and expression level of pro-inflammatory cytokines and NF-κB (30). The connection between the deficiency of Vitamin D and poor asthma outcomes has been previously reported, such as worse symptomatology and poor lung function, and these defects could be reversed for offspring if Vitamin D was supplemented in deficient pregnant rodents (31). Zhen et al. demonstrated that, two out of four VDR polymorphisms could significantly affect the susceptibility of childhood asthma, including FokI and TaqI (8). Similarly, our study supported FokI and TaqI polymorphisms were associated with childhood asthma. Interestingly, it was different from the finding of a previous study (32), which gave support for that VDR gene ApaI (rs7975232) could contribute to asthma susceptibility.

The conflicting results might be explained by the following aspects. Firstly, it is well known that asthma is a clinical syndrome, and no gold standard test have been reported for making the diagnosis. Thus, physicians used multiple algorithms to make the final diagnosis, such as breath shortness, cough history, or wheezing history (33). Meanwhile, other baseline characteristics, such as smoking status, stress, gender, and age, were all related with the diagnosis of asthma (1). Secondly, based on genome-wide analysis studies, the researchers found that over 100 candidate genes were associated with the risk and development of asthma (34). Thirdly, the study designs and different genotyping methods might also account for the conflicting results. The obvious heterogeneity across included studies might also be attributed to these reasons.

There are some limitations should be noted. Firstly, the number of studies included in some subgroups was small, and more high-quality studies would be needed to verify the stability of the results. Secondly, since most of the included studies did not report the family history, living habits and other information of the study subjects, the quantitatively analyze based on these factors could not be performed to determine whether they affect the relationship between VDR gene polymorphisms and the childhood asthma susceptibility. Thirdly, the obvious heterogeneity across included studies could not be ignored. However, the moderate quality suggested that the analysis results had good credibility.

## Conclusion

In summary, we concluded that FokI and TaqI polymorphisms were associated with childhood asthma susceptibility. However, it seems that ApaI and BsmI polymorphisms are not related with childhood asthma susceptibility. Due to these limitations, further multi-center study with high quality should be designed to verify the present conclusion.

## Data Availability Statement

The original contributions presented in the study are included in the article/Supplementary Material, further inquiries can be directed to the corresponding author/s.

## Author Contributions

YZ: conception and design of the research, acquisition of data, analysis and interpretation of data, and drafting the manuscript. SL: statistical analysis and revision of manuscript for important intellectual content. All authors read and approved the final manuscript.

## Funding

This study was supported by the Medical Science and Technology Development project of Yancheng (Nos. YK2019043 and YK2021051).

## Conflict of Interest

The authors declare that the research was conducted in the absence of any commercial or financial relationships that could be construed as a potential conflict of interest.

## Publisher's Note

All claims expressed in this article are solely those of the authors and do not necessarily represent those of their affiliated organizations, or those of the publisher, the editors and the reviewers. Any product that may be evaluated in this article, or claim that may be made by its manufacturer, is not guaranteed or endorsed by the publisher.

## References

[1] Stern J Pier J Litonjua AA. Asthma epidemiology and risk factors. Semin Immunopathol. (2020) 42:5–15. 10.1007/s00281-020-00785-1 32020334

[2] Deng Q Lu C Norback D Bornehag CG Zhang Y Liu W. Early life exposure to ambient air pollution and childhood asthma in China. Environ Res. (2015) 143:83–92. 10.1016/j.envres.2015.09.032 26453943

[3] Makoui MH Imani D Motallebnezhad M Azimi M Razi B. Vitamin D receptor gene polymorphism and susceptibility to asthma, Meta-analysis based on 17 case-control studies. Ann Allergy Asthma Immunol. (2020) 124:57–69. 10.1016/j.anai.2019.10.014 31654764

[4] Wang TT Tavera-Mendoza LE Laperriere D Libby E MacLeod NB Nagai Y. Large-scale in silico and microarray-based identification of direct 1,25-dihydroxyvitamin D3 target genes. Mol. Endocrinol. (2005) 19:2685–95. 10.1210/me.2005-0106 16002434

[5] Amorim C Oliveira JM Rodrigues A Furlanetto KC Pitta F. Vitamin D, association with eosinophil counts and IgE levels in children with asthma. J Bras Pneumol. (2020) 47:e20200279. 10.36416/1806-3756/e20200279 33174974 PMC7889310

[6] Emami Ardestani M Movahedi A. Effect of vitamin D supplementation on improvement of symptoms in mild-to-moderate asthma patients with vitamin d insufficiency and deficiency. Tanaffos. (2020) 19:322–9. 33959169 PMC8088147

[7] Kostner K Denzer N Muller CS Klein R Tilgen W Reichrath J. The relevance of vitamin D receptor (VDR) gene polymorphisms for cancer, a review of the literature. Anticancer Res. (2009) 29:3511–36. 19667145

[8] Zhen YF Wang L. Relationship of vitamin D receptor gene polymorphism with children asthma and wheezy bronchitis (In Chineses). Chin J Prev Vet Med. (2010) 11:1055−8.

[9] Ahmed AE Hassan MH Toghan R Rashwan NI. Analysis of 25-hydroxy cholecalciferol, immunoglobulin E, and vitamin D receptor single nucleotide polymorphisms (Apa1, Taq1, and Bsm1), among sample of Egyptian children with bronchial asthma, A case-control study. Ann Allergy Asthma Immunol. (2020) 55:1349–58. 10.1002/ppul.24785 32311846

[10] Hou C Zhu X Chang X. Correlation of vitamin D receptor with bronchial asthma in children. Exp Ther Med. (2018) 15:2773–6. 10.3892/etm.2018.5739 29456680 PMC5795668

[11] Hutchinson K Kerley CP Faul J Greally P Coghlan D Louw M. Vitamin D receptor variants and uncontrolled asthma. J Clin Lab Anal. (2018) 50:108–16. 10.23822/EurAnnACI.1764-1489.46 29384117

[12] Kilic M Ecin S Taskin E Sen A Kara M. The vitamin D receptor gene polymorphisms in asthmatic children, a case-control study. Pediatr Allergy Immunol. (2019) 32:63–9. 10.1089/ped.2018.0948 31508258 PMC6733045

[13] Zhu L Li JH Ma XB. Study on the relationship between 25-hydroxyvitamin D concentration and its receptor gene polymorphisms and childhood asthma (In Chinese). Experimental and Laboratory Medicine. (2019) 37:897–900.

[14] Wells G Shea B O'Connell D Peterson J Welch V Losos M. The Newcastle-Ottawa Scale (NOS) for Assessing the Quality of Nonrandomised Studies in Meta-Analyses. (2014). Available online at: https://www.ohri.ca/programs/clinical_epidemiology/oxford.asp (accessed 20 June, 2021).

[15] Higgins JP Thompson SG Deeks JJ Altman DG. Measuring inconsistency in meta-analyses. BMJ. (2003) 327:557–60. 10.1136/bmj.327.7414.557 12958120 PMC192859

[16] Batmaz SB Arikoglu T Uyar N Barlas I Kuyucu S. The effect of vitamin D pathway genes on asthma susceptibility, asthma control and vitamin D levels in Turkish Asthmatic children. Int J Hum Genet. (2017) 17:76–85. 10.1080/09723757.2017.1351128

[17] Einisman H Reyes ML Angulo J Cerda J López-Lastra M Castro-Rodriguez JA. Vitamin D levels and vitamin D receptor gene polymorphisms in asthmatic children, a case-control study. Pediatr Allergy Immunol. (2015) 26:545–50. 10.1111/pai.12409 26011658

[18] Iordanidou M Paraskakis E Giannakopoulou E Tavridou A Gentile G Borro M. Vitamin D receptor ApaI a allele is associated with better childhood asthma control and improvement in ability for daily activities. Omics. (2014) 18:673–81. 10.1089/omi.2014.0023 25353337

[19] Ismail MF Elnady HG Fouda EM. Genetic variants in vitamin D pathway in Egyptian asthmatic children, a pilot study. Hum Immunol. (2013) 74:1659–64. 10.1016/j.humimm.2013.08.284 24007655

[20] Liu Y Zhang H Qiao Y. Relationship of vitamin D receptor gene polymorphism and children asthma and wheezy bronchitis (In Chineses). China J Modern Med. (2016) 26:36–9.

[21] Ma JH Tang CC Zhang XH Gao Y Bai H. Correlation between Vitamin D receptor gene polymorphisms and asthma in Chinldren of Hui nationality in Ningxia (In Chinese). Ningxia Medical J. (2014) 36:870–3. 10.13621/j.1001-5949.2014.100870

[22] Maalmi H Sassi FH Berraies A Ammar J Hamzaoui K Hamzaoui A. Association of vitamin D receptor gene polymorphisms with susceptibility to asthma in Tunisian children, a case control study. Hum Immunol. (2013) 74:234–40. 10.1016/j.humimm.2012.11.005 23200756

[23] Mo LY Deng YC Huang CZ Liu JL. Association between polymorphism of vitamin D receptor gene and asthma in Children (In Chinese). Chin J Contemp Pediatr. (2015) 23:742–4. 10.11852/zgetbjzz2015-23-07-21

[24] Papadopoulou A Kouis P Middleton N Kolokotroni O Karpathios T Nicolaidou P. Association of vitamin D receptor gene polymorphisms and vitamin D levels with asthma and atopy in Cypriot adolescents, a case-control study. Multidiscip Respir Med. (2015) 10:26. 10.4081/mrm.2015.304 26346690 PMC4559891

[25] Pillai DK Iqbal SF Benton AS Lerner J Wiles A Foerster M. Associations between genetic variants in vitamin D metabolism and asthma characteristics in young African Americans, a pilot study. J Investigat Med. (2011) 59:938–46. 10.2310/JIM.0b013e318220df41 21613960 PMC3199964

[26] Zhang Y Wang Z Ma T. Associations of Genetic Polymorphisms Relevant to Metabolic Pathway of Vitamin D3 with Development and Prognosis of Childhood Bronchial Asthma. DNA Cell Biol. (2017) 36:682–92. 10.1089/dna.2017.3730 28590769

[27] Zhao HX Chen XR Wu CY Zhuang HN. Study on the correlation of 25-(OH)-VD and Vitamin D receptor gene polymorphism of Children and asthma (In Chinese). Chin Lab Diagno. (2015). 1894–7.

[28] Horner CC Bacharier LB. Diagnosis and management of asthma in preschool and school-age children: focus on the 2007 NAEPP Guidelines. Curr Opin Pulm Med. (2009) 15:52–6. 10.1097/MCP.0b013e32831da8ea 19077706

[29] Ruan Z Shi Z Zhang G Kou J Ding H. Asthma susceptible genes in children, A meta-analysis. Medicine (Baltimore). (2020) 99:e23051. 10.1097/MD.0000000000023051 33157959 PMC7647564

[30] Hall SC Agrawal DK. Vitamin D and Bronchial Asthma, An Overview of Data From the Past 5 Years. Clin Ther. (2017) 39:917–29. 10.1016/j.clinthera.2017.04.002 28449868 PMC5607643

[31] Yurt M Liu J Sakurai R Gong M Husain SM Siddiqui MA. Vitamin D supplementation blocks pulmonary structural and functional changes in a rat model of perinatal vitamin D deficiency. Am J Physiol Cell Physiol. (2014) 307:L859–867. 10.1152/ajplung.00032.2014 25305247 PMC4254963

[32] Zhao DD Yu DD Ren QQ Dong B Zhao F Sun YH. Association of vitamin D receptor gene polymorphisms with susceptibility to childhood asthma, A meta-analysis. Pediatr Pulmonol. (2017) 52:423–9. 10.1002/ppul.23548 27551963

[33] de Jong C. C. M. Pedersen E. S. L. Mozun R. Muller-Suter D. Jochmann A. Singer F.. (2020). Diagnosis of asthma in children, findings from the Swiss Paediatric Airway Cohort. Eur Respir J. 56 10.1183/13993003.congress-2020.4020 32499334

[34] MacArthur J Bowler E Cerezo M Gil L Hall P Hastings E. The new NHGRI-EBI Catalog of published genome-wide association studies (GWAS Catalog). Nucleic Acids Res. (2017) 45:D896–901. 10.1093/nar/gkw1133 27899670 PMC5210590

