# Supplementary material for: Meta-Analysis of Vitamin D Receptor Gene Polymorphisms in Childhood Asthma
Source: Front Pediatr. 2022 Apr 1;10:843691. doi: 10.3389/fped.2022.843691 (PMC9010509; doi:10.3389/fped.2022.843691)
Supplement: Supplementary file 1 [file Data_Sheet_1.docx]

**Sup Table 1 Selection strategy and results in PubMed** (The retrieval time: 20210412)

| Search | Query | Items found |
| --- | --- | --- |
| #1 | ("receptors, calcitriol"[MeSH Terms] OR "calcitriol receptors"[All Fields] OR "vitamin d receptor"[All Fields] OR "VDR"[All Fields]) | 11638 |
| #2 | ("genetic"[All Fields] OR "genetical"[All Fields] OR "genetically"[All Fields] OR "genetics"[MeSH Subheading] OR "genetics"[All Fields] OR "genetics"[MeSH Terms] OR ("polymorphic"[All Fields] OR "polymorphics"[All Fields] OR "polymorphism s"[All Fields] OR "polymorphism, genetic"[MeSH Terms] OR ("polymorphism"[All Fields] AND "genetic"[All Fields]) OR "genetic polymorphism"[All Fields] OR "polymorphism"[All Fields] OR "polymorphisms"[All Fields]) OR ("variant"[All Fields] OR "variants"[All Fields])) | 4506798 |
| #3 | ("asthma"[MeSH Terms] OR "asthma"[tiab]) | 181306 |
| #4 | ("child"[MeSH Terms] OR "child"[All Fields] OR "children"[All Fields] OR "childrens"[All Fields] OR "childs"[All Fields] OR "adolescent"[MeSH Terms] OR "adolescent"[All Fields] OR "teenage"[All Fields] OR "teenager"[All Fields] OR "teenagers"[All Fields] OR "teenaged"[All Fields] OR "teenages"[All Fields] OR "paediatrics"[All Fields] OR "pediatrics"[MeSH Terms] OR "pediatrics"[All Fields] OR "paediatric"[All Fields] OR "pediatric"[All Fields]) | 4165055 |
| #5 | #1 AND #2 AND #3 AND #4 | 54 |

**Sup Table 2 Selection strategy and results in Embase** (The retrieval time: 20210412)

| Search | Query | Items found |
| --- | --- | --- |
| #1 | ('vitamin d receptor'/exp OR 'vitamin d receptor' OR VDR) | 16103 |
| #2 | ('genetic'/exp OR 'genetic' OR 'genetical' OR 'genetically' OR 'genetics'/exp OR 'genetics' OR 'polymorphic' OR 'polymorphics' OR 'genetic polymorphism'/exp OR 'genetic polymorphism' OR 'polymorphism'/exp OR 'polymorphism' OR 'polymorphisms' OR 'variant'/exp OR 'variant' OR 'variants') | 2916324 |
| #3 | ('asthma'/exp OR asthma) | 292813 |
| #4 | ('children'/exp OR children OR 'child'/exp OR child OR 'teenager'/exp OR teenager OR 'pediatric'/exp OR pediatric) | 3404132 |
| #5 | #1 AND #2 AND #3 AND #4 | 104 |
